# P-1203. Acute Respiratory Viral Infections in Children with Malignancy in the CDC New Vaccine Surveillance Network, 2016–2020

**DOI:** 10.1093/ofid/ofae631.1385

**Published:** 2025-01-29

**Authors:** Ruby Sangha, Samar Musa, Jonathan Albert, Janet A Englund, Eileen J Klein, Julie A Boom, Leila C Sahni, Christopher J Harrison, Rangaraj Selvarangan, Natasha B Halasa, Jennifer E Schuster, Geoffrey A Weinberg, Peter G Szilagyi, Mary A Staat, Laura S Stewart, Heidi L Moline, Leah Goldstein, John Williams, Marian G Michaels

**Affiliations:** UPMC Childrens Hospital of Pittsburgh, Pittsburgh, Pennsylvania; University of Pittsburgh, Pittsburgh, Pennsylvania; UPMC Childrens Hospital of Pittsburgh, Pittsburgh, Pennsylvania; Seattle Children’s Hospital, Seattle, Washington; University of Washington School of Medicine, Seattle, Washington; Texas Children’s Hospital, Houston, Texas; Baylor College of Medicine and Texas Children’s Hospital, Houston, Texas; Children's Mercy Hospital, Kansas City, Missouri; Children’s Mercy Kansas City, Kansas City, Missouri; Vanderbilt University Medical Center, Nashville, TN; Children’s Mercy Kansas City, Kansas City, Missouri; University of Rochester School of Medicine & Dentistry, Rochester, NY; UCLA School of Medicine, Agoura Hills, California; Cincinnati Children’s Hospital Medical Center, Cincinnati, Ohio; Vanderbilt University Medical Center, Nashville, TN; Centers for Disease Control and Prevention, Atlanta, Georgia; CDC, Atlanta, Georgia; UPMC Childrens Hospital of Pittsburgh, Pittsburgh, Pennsylvania; UPMC Children's Hospital of Pittsburgh, Pittsburgh, Pennsylvania

## Abstract

**Background:**

Acute respiratory infections (ARI) can be severe in children with malignancy. We studied the frequency of respiratory viruses in children with cancer (leukemia, lymphoma, solid organ, or CNS) who presented with ARI in the CDC New Vaccine Surveillance Network.

**Table:**
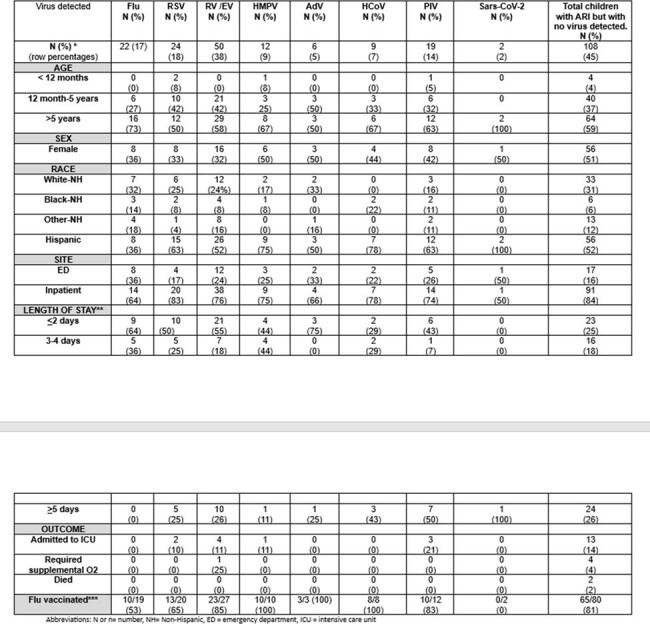
Characteristics of Children with Cancer and Acute Respiratory Illness by Respiratory Virus Detected (December 2016–September 2021), N=240 Abbreviations: N or n= number, NH= Non-Hispanic, ED = emergency department, ICU = intensive care unit *12 co-infections were noted (a total of 144 viruses were isolated) **Applies to inpatients only. *** Flu vaccinated included patients > 6 months of age verified as receiving at least one flu vaccine during the flu season in which the patient was enrolled. We did not examine vaccination timing in relation to illness onset or confounders for this study, and thus did not calculate vaccine effectiveness. The percentages represent the number vaccinated/number eligible for vaccine among patients age > 6 months during flu season. The number and percentages shown for detection of each virus (top row) are row percentages whereas the remaining percentages in the table are column percentage. The (2) SARS-CoV-2 detections were enrolled outside of flu season. *Total of 240 cancer patients, 230 were tested, 179 were eligible for the flu vaccine- those enrolled outside of the flu season or who were too young to receive a flu vaccine were excluded,

**Methods:**

Active, prospective surveillance was conducted for children < 18 in the emergency department (ED) or hospitalized with ARI at 7 children’s hospitals from 12/1/16-9/28/20. ARI was defined as ≥1 of fever, cough, otalgia, congestion, rhinorrhea, sore throat, wheezing, shortness of breath/rapid or shallow breathing, apnea, or brief resolved unexplained event. Febrile neutropenia without ARI was excluded. Nasal/throat swabs were tested for respiratory syncytial virus (RSV), influenza A/B/C(Flu), parainfluenza 1-4(PIV), human metapneumovirus (HMPV), rhinovirus/enterovirus (RV/EV), adenovirus (AdV), and seasonal coronaviruses (HCoV), and SARS-CoV-2 after 1/1/20. Demographic/clinical data were collected by parent interview and chart abstraction. Patients were considered vaccinated against flu if they were > 6 months and had ≥ 1 dose verified from a registry or provider..

**Results:**

Of 34,455 ARI patients enrolled, 240(0.7%) had cancer, with a median age of 7 years and 187(78%) were hospitalized. A virus was detected in 132(55%), with > 1 virus detected in 12(5%). Viruses included RV/EV (38%), RSV (18%), Flu (17%), and PIV (14%) (Table). Inpatient and ICU admissions, respectively, were most common with RSV (83%, 10%), RV/EV (76%, 11%), HMPV (75%, 11%), and PIV (74%, 21%). Prolonged hospitalization ( > 5 days) was more common with PIV (50%), HCoV (43%), and RV/EV (26%). 10/19(53%) flu-positive children were vaccinated compared with 132/160(83%) flu-negative (p< 0.05). Mechanical ventilation was required for 1/50(2%) RV/EV-positive and 4/110(4%) of cancer patients with no virus identified.

**Conclusion:**

Over 50% of children with cancer and ARI tested positive for ≥ 1 virus. Some viruses were associated with higher rates of hospitalization, ICU, and prolonged stay. There was a lower vaccination rate among flu-positive compared to flu-negative children. The study highlights the impact of viral ARI on children with cancer, emphasizing the need for prevention.

**Disclosures:**

**Janet A. Englund, MD**, Abbvie: Advisor/Consultant|AstraZeneca: Advisor/Consultant|AstraZeneca: Grant/Research Support|GlaxoSmithKline: Advisor/Consultant|GlaxoSmithKline: Grant/Research Support|Meissa Vaccines: Advisor/Consultant|Merck: Advisor/Consultant|Pfizer: Board Member|Pfizer: Grant/Research Support|Pfizer: Speaker at meeting|SanofiPasteur: Advisor/Consultant|Shinogi: Advisor/Consultant **Christopher J. Harrison, MD**, GSK: Grant/Research Support|Medscape: Honoraria|Merck: Grant/Research Support|Pfizer: Grant/Research Support|UpToDate: Honoraria **Rangaraj Selvarangan, BVSc, PhD, D(ABMM), FIDSA, FAAM**, Abbott: Grant/Research Support|Abbott: Honoraria|BioMerieux: Grant/Research Support|Cepheid: Grant/Research Support|Diasorin: Grant/Research Support|GSK: Advisor/Consultant|Hologic: Grant/Research Support|Luminex: Grant/Research Support|Qiagen: Grant/Research Support **Natasha B. Halasa, MD, MPH**, Merck: Grant/Research Support **Geoffrey A. Weinberg, MD**, Inhalon: Advisor/Consultant|Merck & Company: Honoraria for textbook chapter preparation **Mary A. Staat, MD, MPH**, Cepheid: Grant/Research Support|Merck: Grant/Research Support|Pfizer: Grant/Research Support|Up-To-Date: Honoraria

